# An ensemble-based machine learning model for predicting type 2 diabetes and its effect on bone health

**DOI:** 10.1186/s12911-024-02540-0

**Published:** 2024-05-29

**Authors:** Belqes Alsadi, Saleh Musleh, Hamada R. H. Al-Absi, Mahmoud Refaee, Rizwan Qureshi, Nady El Hajj, Tanvir Alam

**Affiliations:** 1 College of Science and Engineering, Hamad Bin Khalifa University, Doha, Qatar; 2 Hamad Medical Corporation, Doha, Qatar; 3 Department of Imaging Physics, MD Anderson Cancer Center, The University of Texas, Houston, USA; 4 College of Health and Life Sciences, Hamad Bin Khalifa University, Doha, Qatar

**Keywords:** Machine learning, Diabetes, Bone health, Dual Energy X-ray Absorptiometry, Qatar Biobank (QBB)

## Abstract

**Background:**

Diabetes is a chronic condition that can result in many long-term physiological, metabolic, and neurological complications. Therefore, early detection of diabetes would help to determine a proper diagnosis and treatment plan.

**Methods:**

In this study, we employed machine learning (ML) based case-control study on a diabetic cohort size of 1000 participants form Qatar Biobank to predict diabetes using clinical and bone health indicators from Dual Energy X-ray Absorptiometry (DXA) machines. ML models were utilized to distinguish diabetes groups from non-diabetes controls. Recursive feature elimination (RFE) was leveraged to identify a subset of features to improve the performance of model. SHAP based analysis was used for the importance of features and support the explainability of the proposed model.

**Results:**

Ensemble based models XGboost and RF achieved over 84% accuracy for detecting diabetes. After applying RFE, we selected only 20 features which improved the model accuracy to 87.2%. From a clinical standpoint, higher HDL-Cholesterol and Neutrophil levels were observed in the diabetic group, along with lower vitamin B12 and testosterone levels. Lower sodium levels were found in diabetics, potentially stemming from clinical factors including specific medications, hormonal imbalances, unmanaged diabetes. We believe Dapagliflozin prescriptions in Qatar were associated with decreased Gamma Glutamyltransferase and Aspartate Aminotransferase enzyme levels, confirming prior research. We observed that bone area, bone mineral content, and bone mineral density were slightly lower in the Diabetes group across almost all body parts, but the difference against the control group was not statistically significant except in T12, troch and trunk area. No significant negative impact of diabetes progression on bone health was observed over a period of 5-15 yrs in the cohort.

**Conclusion:**

This study recommends the inclusion of ML model which combines both DXA and clinical data for the early diagnosis of diabetes.

**Supplementary Information:**

The online version contains supplementary material available at 10.1186/s12911-024-02540-0.

## Introduction

Diabetes mellitus is a metabolic disorder characterized by excessive glucose (sugar) levels in the blood that can be controlled with proper diet, exercise, or medications. Diabetes is a common and increasing non-communicable disease with high prevalence rates worldwide. It may also increase the risk of kidney disease, heart disease, blindness, amputation, osteoporosis, etc. [1]. Type 1 diabetes (T1D) is when beta cells in the pancreas stop producing insulin, while Type 2 diabetes (T2D), previously referred to as adult-onset diabetes, occurs when muscle, liver, and fat cells develop resistance to insulin [2]. The number of diagnosed diabetic patients is currently on the rise, and it is one of the most common conditions affecting people of all ages [3]. According to a World Health Organization (WHO), $\sim$ 393 million people were living with diabetes in 2011 [4]. Diabetes statistics from 2013 showed an increase to 415 million diabetic patients worldwide, which indicates that diabetes is rapidly expanding from a widespread health problem to a worldwide epidemic [5]. Diabetes in the leading cause of death in most developed countries, and mounting evidence suggests that it is becoming more common in several developing countries. According to the International Diabetes Federation (IDF), the population with diabetes is projected to increase to 629 million by 2045 [6].

**Fig. 1:**
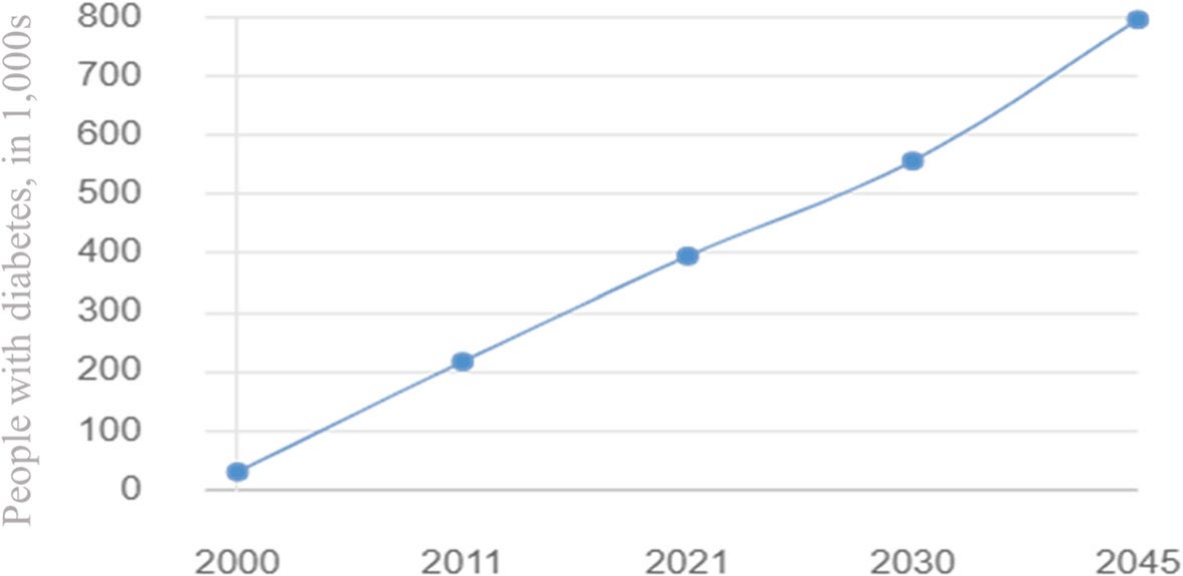
Diabetes status and expected progression report in Qatar 2000 - 2045 [9]

As reported by the Ministry of Public Health in Qatar, diabetes is the leading cause of death in the country causing an economic burden on the healthcare sector. The prevalence of diabetes in Qatar is among the highest in the world and is rising dramatically when compared to regional and international averages. In 2008, the WHO projected that the global prevalence of diabetes among persons aged 25 and older was approximately 10%, with the greatest rates in the Middle East and the Americas (11% for both sexes) [7]. Moreover, The IDF report highlighted that the prevalence of diabetes among adults in Qatar increased from 3% in 1991 to more than 12% in 2000 and later to 17.5% in 2006. The largest increase in diabetes rate was observed for women, with an increase from 4% to 18% [8]. As shown in Fig. 1, the number of people with diabetes in Qatar has been steadily increasing over the past decade, and this increase is expected to continue in the coming years [9].

Multiple factors can affect diabetes, including diet and exercise. The relationship between these two is of particular interest. A study by Hassan et al., compared diabetics vs non-diabetics to understand how physical activity may influence bone health in the Qatari population [10]. Nazeemudeen et al. conducted a study on Qatari diabetic cohort of 500 person to evaluate their food habit and physical activity level [11]. Only a limited number of studies have been conducted in Qatar to predict diabetes using ML techniques. Abbas et al. [12] conducted a study on 7268 Qatari citizens, and their objective was to identify significant risk factors for prediabetes in the Middle East. The results showed great promise in detecting prediabetes early on and, as a result, reducing the incidence of diabetes in the region. Using 2,590 individuals from Qatar Biobank (QBB), Sadek et al. [13] developed two scoring models to identify individuals at risk of developing impaired glucose metabolism (IGM) or type two diabetes mellitus (T2DM). This study evaluated and compared several scoring models for T2DM screening, which lead to the development of a Qatari-specific diabetes and IGM risk scores to identify high-risk individuals and can thus help establish a nationwide primary prevention program [13]. Furthermore, Musleh et al. developed machine learning (ML) models to classify diabetic patients from non-diabetic participants of the QBB [14]. A total of 25 potential risk factors were identified in this study which could be used to distinguish diabetics from non-diabetics. Based on the identified risk factors, HbA1c, Glucose, and LDL-cholesterol were found to be the most influential risk factors [14]. Recently, Islam et al. proposed a deep learning model DiaNet to diagnose diabetes from retinal images only [15]. The proposed model achieved over 84% accuracy in diagnosing Qatari population in the QBB cohort [15]. An update of DiaNet model is recently been published with hither accuracy of 92% [16]. Recently Wachinger et al. proposed a deep learning model for the detection of T2D based on MRI images only [17]. Based on the MRI images the authors achieved an accuracy of 78.7%. Sadek et al. used demographics and anthropometic metasurements for the early detection of diabetes [18]. UK Biobank collection of accelerometer traces from 103712 was used for the T2D detection [19] The proposed model achieved F1-score of around 0.80 for positive class and 0.73 for negative class. Interested readers are referred to this article for a quick review on the existing ML models for controlling diabetes [20, 21]. A summary of the ML based studies for diabetes detection is presented in Table 1.

**Table 1:** A summary of previous articles that focus on machine-learning algorithms for diagnosing diabetes. QBB: Qatar Biobank

Reference	Year	Cohort Size	Cohort Summary	Remarks
[13]	2018	2590	From QBB, 1660 participants were selected for training models and 930 participants for validation	To develop two scoring models for identifying Qatari individuals at risk for developing impaired glucose metabolism (IGM) or type two diabetes mellitus (T2DM).
[14]	2020	3200	The data were obtained from QBB	A comprehensive analysis of dataset including anthropometric data, medical tests, spirometry measurements,etc. This study identified key risk factors associated with diabetes that are likely to be a contributing factor in the Qatari population using ML techniques.
[15]	2020	500	Qatari adult population from QBB	Retinal image-based diabetes diagnosis. A deep learning-based model, DiaNet, was proposed to diagnose diabetes from retinal image only.
[11]	2020	500	Adult Qatari citizen	Statistical analysis on food habit shows they consume higher level of sugar in tea and need to improve physical activity level.
[12]	2021	7268	Adult controls and prediabetic adults from the QBB	Logistic regression and other ML models were used to develop a risk score to detect prediabetes in the Middle East.
[18]	2021	2000	QBB collection of participants for their demographics and anthropometric measurements	Gender, age, waist-to-hip-ratio, history of hypertension were statistically significant in detecting diabetes.
[19]	2021	103712	UK Biobank collection of accelerometer traces	Accelerometer traces was used for diabetes detection.
[17]	2023	3406	Participants from UK Biobank	MRI image was used for diabetes detection.
[16]	2024	5545	Adult participants from QBB and HMC hospital	Retinal image-based VGG-11 model for diabetes diagnosis.

Diabetes can have lifelong consequences on your physical health, including influencing the bone health. Bone mineral density provides one measure of how well the bones are working and lower bone mineral density may be associated with a higher risk for fractures when patients become older [22]. Dual X-ray Absorptiometry (DXA) measures body composition in a non-invasive and fast manner [23] in terms of mass, fat, bone, and muscle composition. Because of its reliability and accuracy, DXA has become the gold standard for measuring bone mass and overall body composition [23]. Recently Musleh et al. used DXA data to analyze the bone health of the QBB diabetic cohort and build a model on early onset of osteoporosis or osteopenia [24]. ML-based technique has recently been proposed to find the link between DXA and cardiovascular disease [23]. This study aims to develop ML for identifying diabetic and non-diabetic patients in Qatar using two different types of datasets collected from the QBB dataset. The first dataset focuses on the bone health indicators derived from full-body DXA scan measurements, whereas the second dataset includes the clinical lab results based on the blood samples. The contribution of this thesis can be summarized as follows:

1. We proposed an ML-based model based on DXA and clinical data for the early detection of diabetes in a cohort size of 1000 from QBB.
2. The proposed model achieved over 87% accuracy in identifying diabetes patients from normal participants even without considering the known biomarkers such as glucose and HbA1c leading towards the discovery of potential novel biomarker for diabetes. Moreover, we showed that combination DXA with clinical data improved the performance of ML model.
3. Our study revealed that the control group exhibited greater bone area, BMC, lean mass, fat mass, and bone mass for almost all body parts in comparison to the target group. But we could not observe any deteriorating effect of diabetes progression on bone health of diabetic patients over a period of 5-15yrs of time.

The article is organized in following sections. In Material and methods section, we have provided a high-level summary of overall method with a schematic diagram. Then we provided details of the dataset used in the study. We also provided details of statistical analysis and machine learning (ML) model development workflow. In Results section, we have provided the results from statistical analysis as well as the performance of ML models. In Discussion section, we highlighted the principal findings of the work, compared the performance of the proposed ML model against other existing models, and limitation of the study. Then in the Conclusion and future works section, we conclude with the future works and final remarks of this work.

## Material and methods

**Fig. 2:**
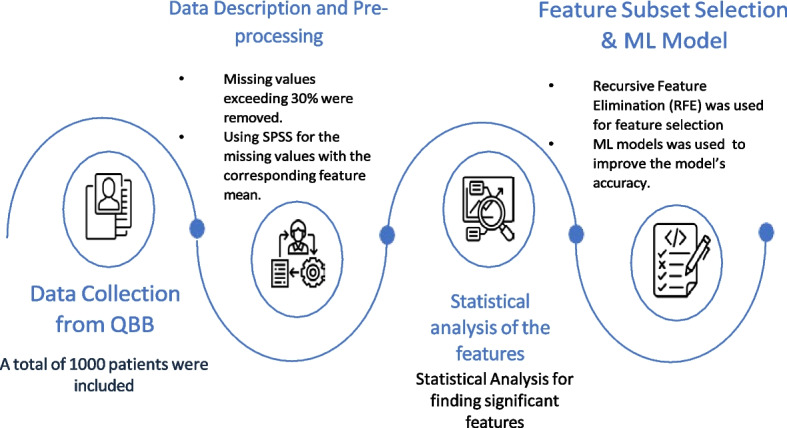
Overall summary of the workflow for this study

In this case-control study, we first collected clinical information from the QBB participants. Then data preprocessing steps were applied to clean the dataset. ML models were developed to distinguish diabetes patients from the control group highlighting that there exists significant difference in the clinical profile of these two groups. To understand the difference of their profile and identify key biomarkers that distinguish the groups, we used statistical technique, RFE based feature subset selection. Moreover, we used SHAP to quantify the relative importance of the proposed markers for detecting diabetes from normal cases. Figure 2 highlights the schematic diagram of the workflow adopted for this study.

### Data collection from QBB

In this study, we collected deidentified data from QBB for a cohort of 500 participants with the type 2 diabetes (T2D) having HbA1c >6.5. As part of our study, we had a group of 500 non-diabetic participants (HbA1c $\le$ 6.5) who were free from diabetes. A total of 1000 participants from QBB were included in the study, of which 541 were males and 459 were females. In the diabetic group there were 209 males and 291 females. The study protocol was approved by IRB committee of QBB (according to the guidelines of the Ministry of Public Health, Qatar) and only de-identified dataset was obtained from QBB.

### Data description and pre-processing

The dataset contained 163 different measurements from DXA. In DXA machines, different body parts are scanned for densitometry and composition. Densitometry measures bone Area, weight, height, bone mineral content (BMC), and bone mineral density (BMD). DXA composition measurement measures bone mass, fat mass, and lean mass. The dataset also includes lab results for QBB participants based on their blood samples. Measurements having missing values exceeding 30% of total records were removed. For the remaining measurements, we replaced the missing values by the corresponding feature mean using PASW Statistics 18 (SPSS Inc.). Finally, 129 features from DXA and 77 features from clinical data were obtained for analysis. It is important to emphasize that we dropped measurements like glucose level, HbA1c for building ML models as these known biomarkers would bias the outcome of ML model.

### Statistical analysis of the features

Statistics were analysed using JASP software. Both the target and control groups were analysed by descriptive statistics. Moreover, all data were subjected to a normality test to ensure that they were distributed normally. We used the student t-test and Mann-Whitney U (MU) test to determine the significance level for the target and control groups.

### Feature subset selection

As part of the development of ML models with highly relevant features, feature subset selection (FSS) technique was employed to select a subset of key features. In the FSS technique, information is eliminated without significant loss by eliminating redundant or highly correlated features from the dataset [25]. In this study, we applied Recursive Feature Elimination (RFE) to enhance the generalization capability of the model by decreasing its variance. Due to its simplicity and effectiveness, this algorithm selects the features (columns) in a training dataset that have greater or lesser relevance to predicting the target variable within a training dataset [25].

### Machine learning model development, evaluation and explnation

Our research objective was to develop ML models to distinguish diabetic patients from non-diabetic people using clinical measurements from blood sample and DXA scan measurements. The following ML algorithms were used: Logistic Regression (LR), Support Vector Machine (SVM), Decision Tree (DT), Random Forest (RF), Naive Bayes (NB), k-Nearest Neighbor (KNN), Artificial Neural Network (ANN), XGBoost and CatBoost. A five-fold cross validation was applied to the model to evaluate its performance. For the evaluation of the proposed ML models, we carried out 5 fold cross validation (CV) using 80% of the data as a training dataset and 20% as a testing dataset. The models were evaluated on different testing datasets for every fold. Subsequently, the performance metrics were averaged across all folds to derive the final results. Multiple evaluation metrics (Eqs. 1 - 5) were applied: (1) Accuracy, (2) Sensitivity (Recall), (3) Specificity, (4) Precision, and (5) Matthew’s Correlation Coefficient (MCC) when analysing the performance of ML models:

1 $$\begin{aligned} ACC=\frac{(tp+tn)}{(tp+tn+fp+fn)} \end{aligned}$$

2 $$\begin{aligned} SEN=\frac{tp}{tp+fn} \end{aligned}$$

3 $$\begin{aligned} SPE=\frac{tn}{tn+fp} \end{aligned}$$

4 $$\begin{aligned} Precision= \frac{tp}{tp+fp} \end{aligned}$$

5 $$\begin{aligned} MCC = \frac{tp\cdot tn-fp\cdot fn}{\sqrt{(tp+fp)(tp+fn)(tn+fp)(tn+fn)}} \end{aligned}$$

Here, TP stands for true positive, FN stands for false negative, FP stands for false positive, while TN stands for true negative. Since the dataset was balanced (500:500 for diabetics and non-diabetics), accuracy was used as the evaluation metric to select the final model. All hyperparameters of the models were optimized using GridSearchCV of Scikit-Learn package of Python. For explaining the relative importance of the selected features on the performance of ML models we used PCA Biplot and SHAP [26] analysis.

## Results

### Features with statistical significance

There was a total of 206 features for each participant of the QBB dataset including 129 DXA measurements from seven different body parts and 77 clinical features. The results of analysing all 206 features are shown in Table 2. A total of 31 features were considered as statistically significant ( based on *p*-value $\le$ 0.05) while 173 features were not statistically significant. A detailed analyses of all the features is presented in the Supplementary Table S1 along with their mean, standard deviation, and *p*-values. Out of these 31 features, 4 features were from DXA, 27 features were from clinical measurements (Table 2).

**Table 2:** Summary of the significance Features; Class 1: Diabetic; Class 0: Non-diabetic

Feature	Type	Mean in Diab	STD in Diab	Mean in Control	STD in control	*p*val (t-test)	*p*val (MU)
DT_AREA_TROCH	Bone Area	13.543	2.57	14.001	0.006	0.018	
DT_AREA_T12	Bone Area	10.474	1.53	10.669	1.546	0.046	0.054
DT_AREA_TRUNK	Bone Area	738.456	100.51	749.37	89.511	0.07	0.049
DT_AVG_WIDTH_T12	Anthrothropometric	3.693	0.36	3.743	0.347	0.026	0.019
HANDGRIP_OUT_LEFT	Anthrothropometric	30.039	10.94	32.859	12.52	$1.580 \times 10^{-4}$	0.002
HDL-Cholesterol	clinical	1.37	0.395	1.3	0.378	0.004	0.002
Sodium	clinical	139.59	2.529	140.12	2.306	$5.567 \times 10^{-4}$	0.002
Urea	clinical	4.532	1.466	4.221	1.137	$1.914 \times 10^{-4}$	0.002
Monocyte Auto %	clinical	7.347	1.903	7.683	2.006	0.007	0.003
Neutrophil Auto #	clinical	3.801	1.504	3.571	1.463	0.014	0.014
Vitamin B12	clinical	284.527	148.163	320.606	307.276	0.018	0.016
Basophil Auto %	clinical	0.7	0.371	0.647	0.325	0.016	0.022
Testosterone Total	clinical	9.421	8.363	10.721	9.169	0.019	0.035
Neutrophil Auto %	clinical	54.045	9.206	52.557	9.985	0.014	0.044
ET_OUT_CALC_MAXHR	ExterciseTest	158.336	26.011	177.585	4.825	$5.720 \times 10^{-53}$	$3.606 \times 10^{-63}$
ET_OUT_PLANNED_RUN_TIME	ExterciseTest	510.727	186.276	555.5	160.437	$5.024 \times 10^{-5}$	0.014
HANDGRIP_OUT_RIGHT	Anthrothropometric	31.812	10.84	33.601	12.109	0.014	0.044
HEIGHTWEIGHT_OUT_SITTING_HEIGHT	Anthrothropometric	91.013	14.807	86.809	4.836	$2.227 \times 10^{-9}$	0.038
BP_OUT_SYSTOLIC_BP_Avg	clinical	119.994	14.269	113.118	12.362	$1.133 \times 10^{-15}$	$1.225 \times 10^{-15}$
Chloride	clinical	100.354	2.682	102.288	2.284	$2.317 \times 10^{-32}$	$1.255 \times 10^{-29}$
BP_OUT_CALC_AVG_SYSTOLIC_BP	clinical	119.878	14.681	112.77	12.413	$4.335 \times 10^{-16}$	$1.301 \times 10^{-15}$
Bicarbonate	clinical	26.283	2.361	25.496	2.441	$2.600 \times 10^{-7}$	$1.391 \times 10^{-6}$
BP_OUT_CALC_AVG_DIASTOLIC_BP	clinical	71.186	9.555	68.428	9.303	$4.249 \times 10^{-16}$	$1.477 \times 10^{-5}$
HIPWAIST_OUT_HIPS_SIZE	clinical	100.005	5.366	107.01	9.848	$1.271 \times 10^{-40}$	$1.825 \times 10^{-36}$
Albumin	clinical	43.636	3.568	42.648	3.643	$1.610 \times 10^{-5}$	$1.834 \times 10^{-5}$
BP_OUT_DIASTOLIC_BP_Avg	clinical	71.262	9.19	68.745	9.267	$1.772 \times 10^{-5}$	$3.707 \times 10^{-5}$

### An ablation study based on different types of features used in ML model

Our aim was to assess the effectiveness of the two diverse types of features proposed for developing ML models. An ablation study was conducted on the combination of two types of features, and then we evaluated how ML performed in this combination. Table 3 compares the performance of ML model on different types of features, 129 features are from DXA data, and 77 features are from to clinical data. This study indicates that the LR-based model is accurate in calculating bone area by 69%, whereas the kNN model reaches a score of 56% for Anthropometric measurements, SVM scores 57% for BMC, kNN scores 54% for BMD, KNN scores 55% for bone mass, NB scores 54% for fat mass, and kNN scores 52.6% for lean mass. RF-based and XGBoost models achieved 84.4% accuracy based on all DXA measurements (129 features). The CatBoost model achieved 84.8% accuracy for all 77 features of the Clinical Data.

**Table 3:** Ablation study on ML model performance considering different types of features

Feature Type	No. of Features	Model	ACC	Sen	Spe	Pre	MCC
Bone Area	28	LR	0.692	0.74	0.72	0.67	0.39
		SVM	0.688	0.74	0.72	0.66	0.38
		DT	0.636	0.67	0.66	0.65	0.27
		RF	0.684	0.7	0.64	0.74	0.36
		NB	0.628	0.65	0.58	0.67	0.25
		KNN	0.628	0.67	0.64	0.62	0.26
		XGBoost	0.66	0.64	0.65	0.67	0.32
		CatBoost	0.688	0.68	0.71	0.67	0.38
		ANN	0.696	0.645	0.566	0.710	0.392
Anthropometric measurements	22	LR	0.532	0.6	0.68	0.4	0.09
		SVM	0.52	0.6	0.72	0.35	0.08
		DT	0.524	0.57	0.57	0.51	0.05
		RF	0.488	0.54	0.61	0.4	-0.01
		NB	0.472	0.52	0.62	0.35	-0.03
		KNN	0.556	0.61	0.61	0.51	0.12
		XGBoost	0.512	0.5	0.48	0.55	0.03
		CatBoost	0.54	0.52	0.52	0.56	0.08
		ANN	0.548	0.562	0.465	0.578	0.097
Bone mineral content (BMC)	25	LR	0.532	0.61	0.69	0.4	0.09
		SVM	0.572	0.65	0.7	0.46	0.17
		DT	0.532	0.57	0.47	0.54	0.06
		RF	0.556	0.61	0.7	0.51	0.12
		NB	0.476	0.51	0.2	0.71	-0.11
		KNN	0.52	0.56	0.5	0.54	0.04
		XGBoost	0.492	0.48	0.44	0.55	-0.01
		CatBoost	0.516	0.5	0.52	0.51	0.03
		ANN	0.496	0.554	0.442	0.553	0.047
Bone mineral density (BMD)	25	LR	0.504	0.56	0.61	0.42	0.02
		SVM	0.52	0.58	0.61	0.45	0.05
		DT	0.484	0.53	0.53	0.52	-0.04
		RF	0.48	0.53	0.58	0.43	-0.03
		NB	0.468	0.51	0.41	0.51	-0.07
		KNN	0.54	0.59	0.59	0.5	0.09
		XGBoost	0.508	0.49	0.47	0.55	0.02
		CatBoost	0.488	0.47	0.47	0.51	-0.02
		ANN	0.516	0.488	0.535	0.570	0.0355
Bone Mass	7	LR	0.48	0.53	0.61	0.37	-0.02
		SVM	0.484	0.54	0.61	0.38	-0.01
		DT	0.54	0.59	0.54	0.53	0.08
		RF	0.544	0.59	0.61	0.53	0.09
		NB	0.516	0.58	0.68	0.38	0.06
		KNN	0.552	0.6	0.57	0.54	0.11
		XGBoost	0.508	0.49	0.49	0.53	0.02
		CatBoost	0.516	0.5	0.5	0.54	0.03
		ANN	0.496	0.455	0.488	0.512	0.066
Fat Mass	15	LR	0.484	0.53	0.56	0.42	-0.02
		SVM	0.488	0.54	0.56	0.42	-0.01
		DT	0.456	0.5	0.45	0.48	-0.09
		RF	0.508	0.56	0.56	0.43	0.03
		NB	0.544	0.56	0.32	0.74	0.06
		KNN	0.488	0.54	0.54	0.45	-0.02
		XGBoost	0.476	0.46	0.48	0.47	-0.05
		CatBoost	0.48	0.47	0.42	0.55	-0.04
		ANN	0.46	0.521	0.388	0.479	-0.078
Lean Mass	7	LR	0.476	0.53	0.6	0.38	-0.03
		SVM	0.476	0.52	0.58	0.39	-0.03
		DT	0.508	0.55	0.48	0.51	0.02
		RF	0.48	0.53	0.57	0.37	-0.02
		NB	0.5	0.57	0.68	0.35	0.03
		KNN	0.516	0.57	0.68	0.45	0.05
		XGBoost	0.46	0.44	0.47	0.45	-0.08
		CatBoost	0.456	0.44	0.47	0.44	-0.09
		ANN	0.516	0.479	0.566	0.471	0.029
All DXA features	129	LR	0.828	0.83	0.84	0.82	0.66
		SVM	0.824	0.82	0.83	0.82	0.65
		DT	0.752	0.73	0.74	0.77	0.5
		RF	0.844	0.81	0.78	0.88	0.69
		NB	0.66	0.64	0.64	0.68	0.32
		KNN	0.712	0.7	0.71	0.71	0.42
		XGBoost	0.844	0.82	0.81	0.88	0.69
		CatBoost	0.832	0.81	0.81	0.85	0.66
		ANN	0.616	0.636	0.652	0.654	0.272
All clinical features	77	LR	0.824	0.81	0.82	0.83	0.65
		SVM	0.82	0.81	0.82	0.82	0.64
		DT	0.796	0.77	0.74	0.83	0.59
		RF	0.836	0.82	0.82	0.84	0.67
		NB	0.76	0.73	0.71	0.81	0.52
		KNN	0.724	0.74	0.78	0.67	0.45
		XGBoost	0.844	0.81	0.81	0.88	0.69
		CatBoost	0.848	0.83	0.83	0.87	0.7
		ANN	0.816	0.81	0.837	0.818	0.631

**Fig. 3:**
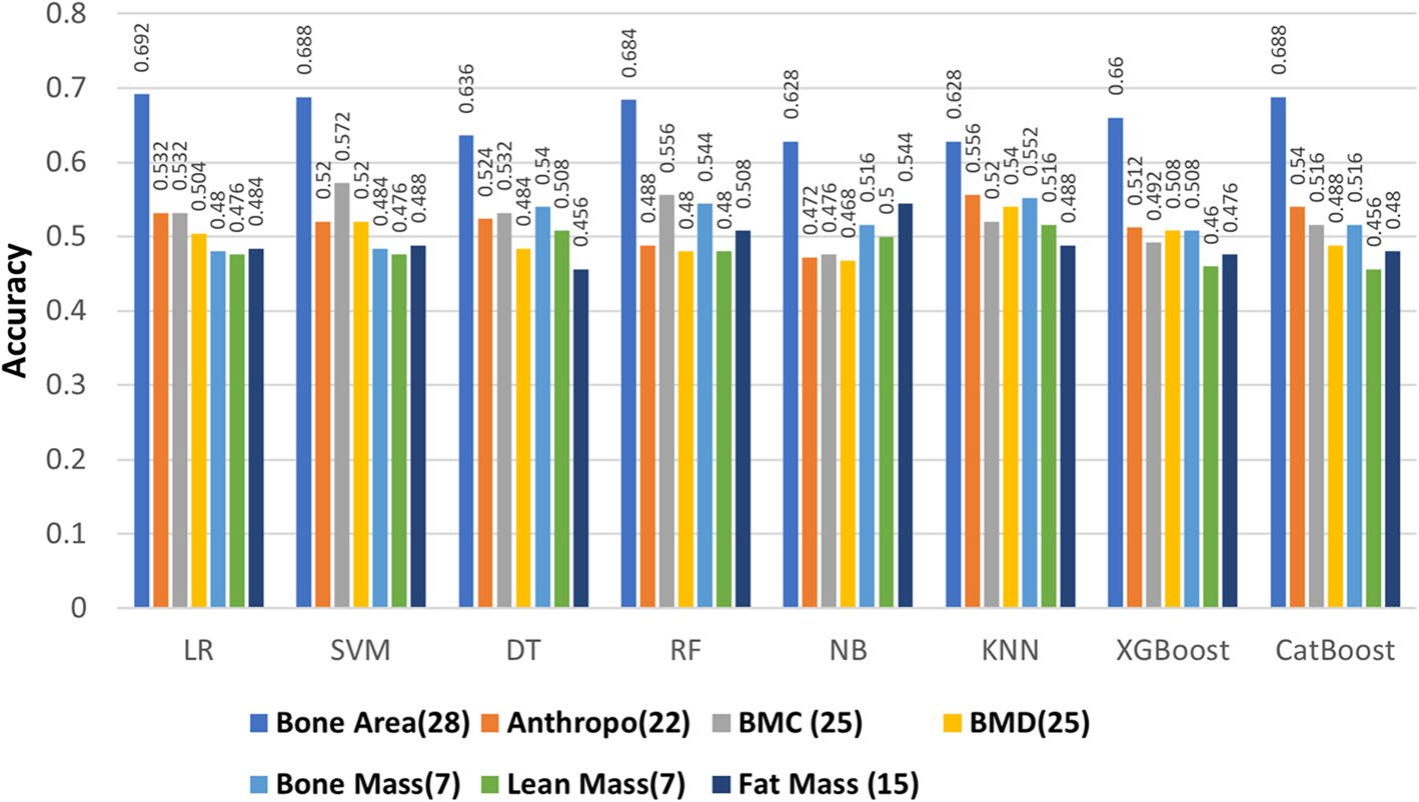
Performance of different ML models on DXA measurements

**Fig. 4:**
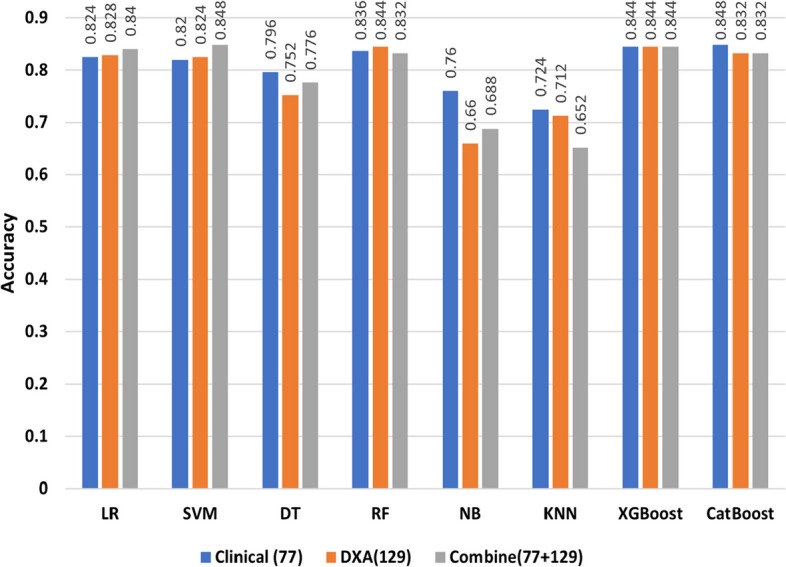
Performance of different ML models on DXA measurements, Clinical data and their combination

In Fig. 3, we compared different types of DXA measurements by feeding them into ML models as different feature groups. We can observe the same performance in different ML models on DXA measurement and bone area with 28 features having the highest performance across all ML algorithms. As further step we combined the features of DXA (129) and clinical data (77), where SVM model had the highest accuracy of 84.8% (Table 4). Most of the models gave better results for clinical data than DXA as shown in Fig. 4, with the exception of RF model in which DXA had better results than clinical data. In addition, the models performed better when clinical data and DXA data were combined.

**Table 4:** Performance of ML model using combination of DXA and clinical features (n=206)

Model	ACC	Sen	Spe	Pre	MCC
LR	0.840	0.881	0.868	0.816	0.682
SVM	0.848	0.889	0.877	0.824	0.698
DT	0.796	0.828	0.789	0.743	0.556
RF	0.882	0.885	0.895	0.794	0.669
NB	0.688	0.615	0.851	0.551	0.416
KNN	0.692	0.702	0.684	0.625	0.308
XGBoost	0.844	0.815	0.914	0.876	0.690
CatBoost	0.832	0.811	0.914	0.851	0.665
ANN	0.816	0.81	0.837	0.818	0.631

### Performance of the model after RFE based feature subset selection

To distinguish diabetic patients from non-diabetic participants, we built different classifiers based on the selected features after RFE. There were 16 features selected from LR and 11 features selected from SVM. We then selected the union of these features. Then RFE based 20 features were used again to run the models. Based on the selected features we found that accuracy levels have increased, with CatBoost achieving the highest accuracy at 87.2% (Table 5).

**Table 5:** Performance of the models after RFE selected features (n=20)

Model	ACC	Sen	Spec	Pre	MCC
LR	0.836	0.819	0.847	0.847	0.672
SVM	0.848	0.840	0.850	0.850	0.700
DT	0.748	0.737	0.752	0.7520	0.4960
RF	0.860	0.830	0.890	0.890	0.720
NB	0.825	0.757	0.874	0.874	0.655
KNN	0.820	0.870	0.770	0.880	0.650
XGBoost	0.812	0.810	0.800	0.910	0.710
CatBoost	0.872	0.831	0.931	0.920	0.750
ANN	0.804	0.795	0.808	0.808	0.600

### Bone health in the QBB diabetic cohort vs. control

Bone area, bone mass, lean mass, and fat mass were measured in both the diabetic (target) and control groups. Almost everywhere on the body, the control group had slightly greater bone area than the target group (Supplementary Table S1). Similarly, we noticed that the control group had slightly higher bone mass, lean mass, fat mass than the diabetes group in all body areas but none of the variables were not statistically significant (Supplementary Table S1). Bone area, bone mass, lean mass, and fat mass were measured in both the diabetic (target) and control groups. Almost everywhere on the body, the control group had slightly greater bone area than the target group (Supplementary Table S1). Similarly, we noticed that the control group had slightly greater bone mass, lean mass, and fat mass than the diabetes group in all body areas but none of the variables were not statistically significant (Supplementary Table S1).

In addition, we noticed a similar trend in other bone health parameters between the diabetes and control groups. We found only three variables representing bone health which are statistically significant while comparing diabetes vs. the control group. Average width of T12 bone, which sits above the lumbar spine, is lower in diabetic group compared to the control group (diab: control = 10.474±1.532: 10.669± 1.546, *p*-value=0.046). The other two significant variables were the area of troch and trunk. And in both of these areas the average area of troch (diab:control = 13.543±2.567: 14.001±2.664, *p*-value=0.006) and trunk (diab:control = 738.456±100.509: 749.37±89.511, *p*-value=0.049) were lower in the diabetic group compared to the control group.

### Impact of diabetes progression on bone health

**Fig. 5:**
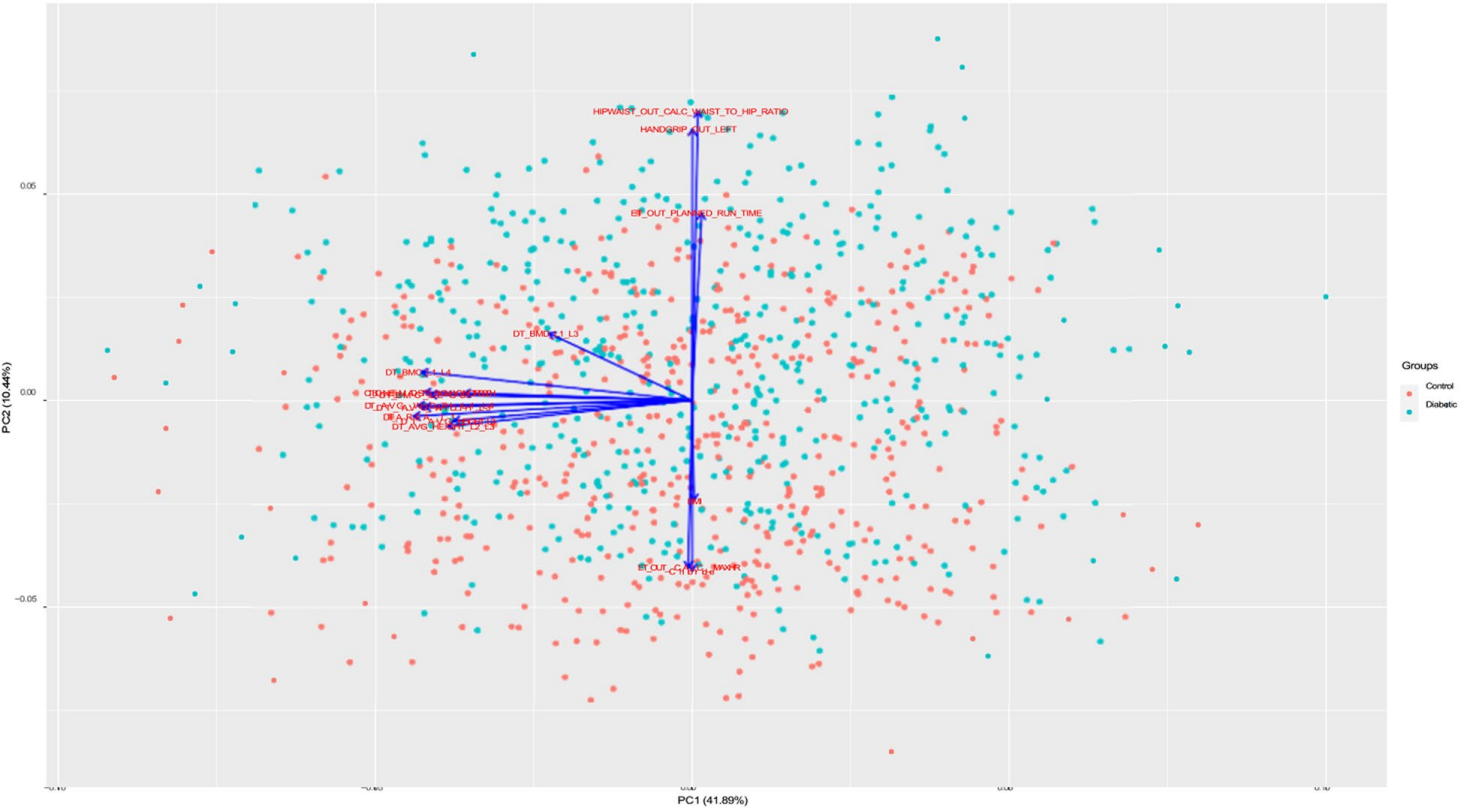
Distribution of Total BMD in participants having diabetes for less than n yrs vs. more than n yrs (n=5,10,15)

Figure 5 shows the distribution on total BMD among diabetes patients who are having diabetes for 5, 10, or 15 yrs. We could not observe any major deteriorating effect of diabetes progression on total BMD over the period of time for diabetic patients (Fig. 5). Rather, in all cases (n=5,10 and 15) we found that the mean value of total BMD was higher for patients having diabetes for a longer period of time (*p*-value = 0.005, 0.012, 0.019 for 5, 10, 15 yrs, respectively).

### Clinical implications

We observed that among the clinical markers HDL-Cholesterol (diab:control = 1.37 ± 0.395 : 1.3$\pm$ 0.378; *p*-value=0.002) and Neutrophil (diab : control= 54.045 ± 9.206: 52.557 ± 9.985; *p*-value=0.044) were having higher values in the diabetic vs. control group in the QBB cohort (Supplementary Table S1). HDL-cholesterol supports to have a better heart health and Neutrophil support to boost the immune system in human. Therefore, these two markers indicating better cardiac health and immune system for the diabetic cohort in Qatar. Higher value of HDL might be due to the fact that diabetic patients in Qatar were taking lipid lowering agent that may contribute to increasing HDL level whis is part of their mechanism of action. These agents lower LDL cholesterol levels, but raise HDL levels [27]. In addition, we observed that vitamin B12 (diab:control= 284.527±148.163: 320.606±307.276; *p*value= 0.018) was lower in the diabetic group since many diabetic patient are on Metformin for controlling blood sugar and this medication may lower vitamin B12 [28]. We also observed lower testosterone levels (diab:control= 9.421±8.363: 10.721±9.169; *p*value= 0.019) in the diabetic group. Many studies have reported a possible link between low testosterone levels and T2D [29].

From the other statistically significant clinical variables, we found Sodium (diab : control= 139.59 ± 2.529 : 140.12 ± 2.306; *p*value= 0.002), Bilirubin (diab : control= 7.931 ± 4.536: 8.468±4.715; *p*value= 0.044), AST (diab:control= 19.084 ± 9.832: 19.43 ± 7.978; *p*value= 0.039), GGT (diab:control = 31.403 ± 27.771 : 35.13 ± 41.018; *p*value= 0.048), etc. we slightly lower in the diabetes group compared to the control group. Low sodium levels, also known as hyponatremia, may result from various factors such as excessive fluid intake, certain medications, hormonal imbalances, and underlying medical conditions. Severe cases of hyponatremia can be seen in people with uncontrolled diabetes who are also experiencing other health complications [30]. Gamma glutamyltransferase (GGT), aspartate aminotransferase (AST), are common liver enzymes and abnormal levels of these enzymes may signal liver function disorder [31]. In Qatar, as many diabetic patients are prescribed dapagliflozin, the decreased levels of these enzymes validate the findings from earlier studies conducted on the Qatari cohort [32].

**Fig. 6:**
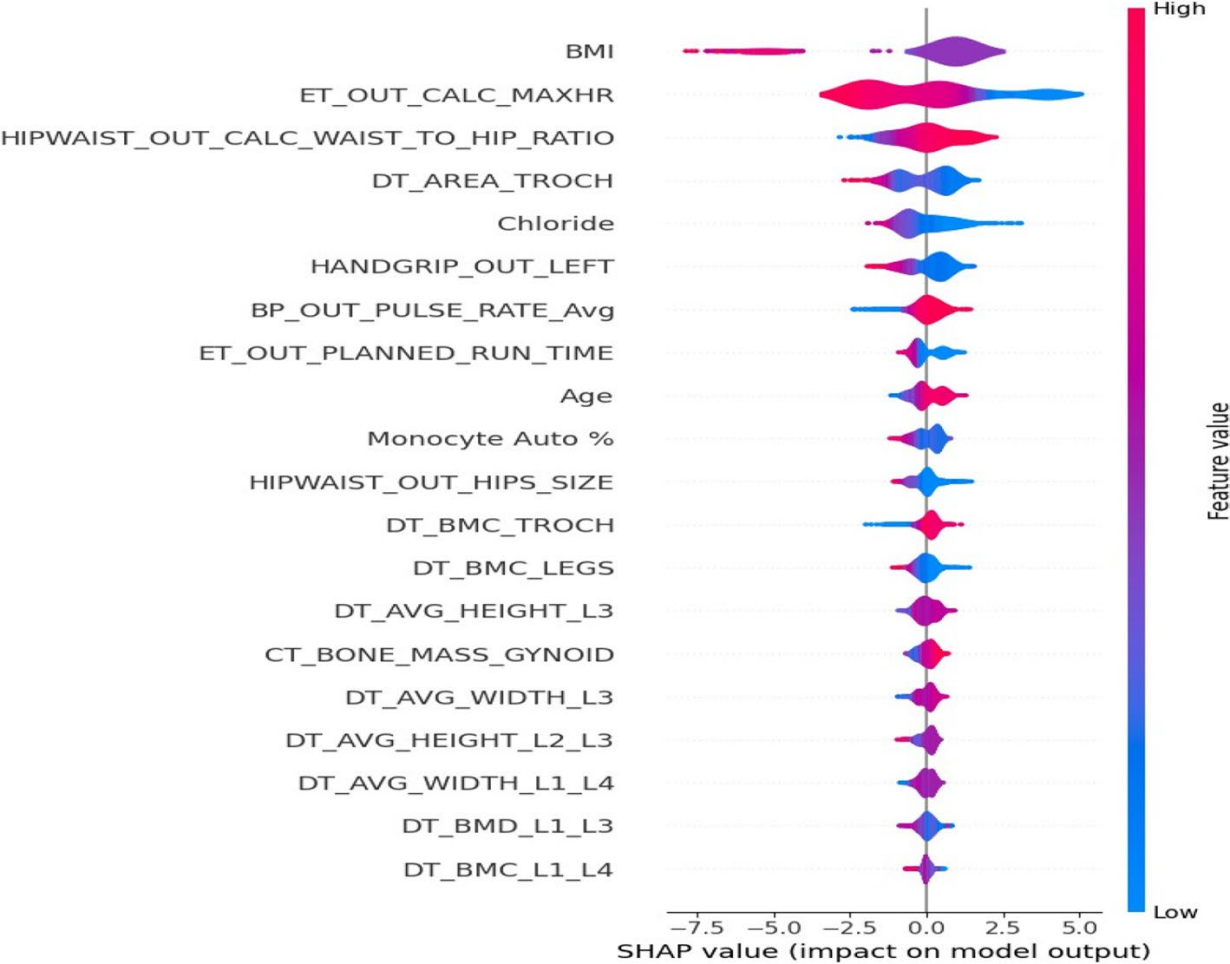
PCA biplot for the selected features by RFE

**Fig. 7:**
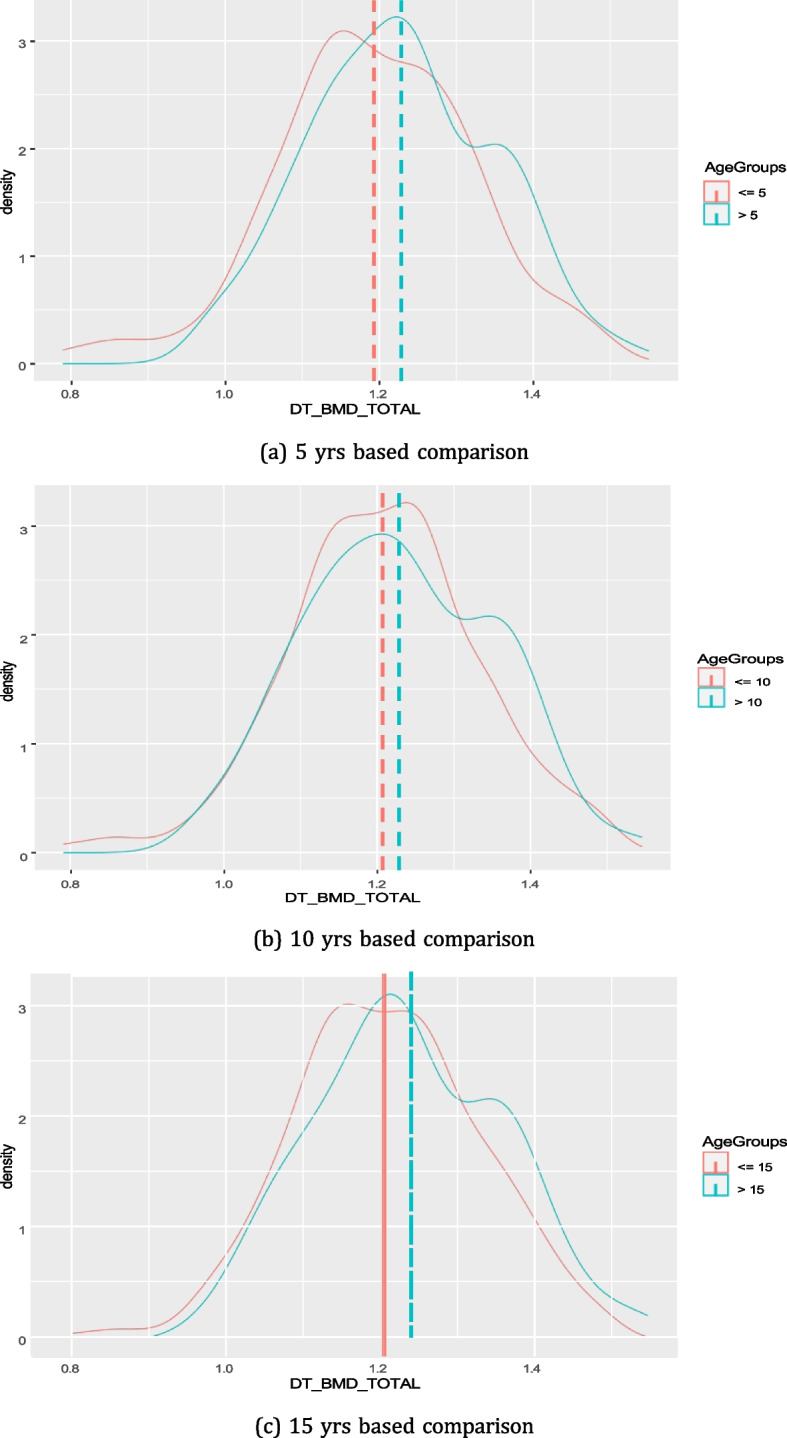
SHAP plot for the selected features by RFE

Figure 6 shows the PCA Biplot for the selected features by RFE. From biplot we can observe that the first two components of the selected features cover over 40% of the variance in the dataset. The direction of vector in Fig. 6 indicates the high correlation between BMI, Chloride and hip circumference. We also observed a nearly opposite direction between chloride and Exercise Test Planned run time. From SHAP analysis of the selected features (Fig. 7), we can observe that BMI, Waist to hip ratio were among the top two important variables for the detection of diabetes. This indicates that obesity plays a big role in diabetes. Lower values of exercise test (“$ER\_OUT\_CALC\_MAXHR$”) for diabetic group indicates that this group need to improve their physical level. From SHAP plot, we also observed the importance of bone densitometry in lumber spines region i.e., L1,L2,L3 and L4,in diagnosing the diabetic patients and their bone health.

## Discussion

In this article, we propose a ML-based approach to predict diabetics from non-diabetics based on a dataset collected from QBB. To develop this model, we used DXA measurements and clinical data. In the following section, we will highlight and discuss the principal findings, compare our methods against other methods, and articulate the usefulness, implications, and limitations of our models.

### Principal findings on ML modelling

In this work, an accuracy of $\ge$ 87% achieved with the proposed ML model for distinguishing diabetic patients from non-diabetic participants. We found that DXA and clinical data can be used to identify diabetics at an early stage. We analysed eight distinct ML models to develop a classifier to differentiate the target group from the control group. Different types of DXA measures were fed into ML models as individual feature groups in an ablation study to determine which ones were most effective. As indicated in Fig. 3, ablation study on different types of DXA measurements showed relatively low accuracy, however bone area showed relatively better accuracy in classifying the diabetes group from the control group with nearly 70% accuracy. When we combined all types of DXA measurements (129 features) in the models, the performance of the models improved to reach $\ge$84% accuracy. Among all the models, RF and XGBoost attained the highest accuracy of $\ge$ 84.4%. For 77 clinical data features, the performance of the models was better compared to the individual type of DXA features (Figs. 3 and 4). Boosting-based algorithms such as XGBoost and CatBoost were among the top-performing algorithms. With an accuracy of 84.8%, CatBoost achieved the best performance among all the models we evaluated. Finally, when all the DXA features and clinical data were combined to build ML models, it achieved the best performing model (Fig. 4). As shown in Fig. 4, the performance of the models based on the combination of DXA and clinical features achieved the best performance accuracy for SVM (84.8%), XGBoost (84.4%) and CatBoost (83.2%). It is important to emphasize that introducing complex model such as ANN than simpler model i.e., LR does not guarantee a higher performing results as evident in Tables 3, 4, and 5. The performance of model depends upon the dataset we are working on and the underlying pattern that model can discover out of this approach. After applying RFE, we obtained a shorter list of selected features, which were used to re-run the models. The results indicated that 16 features were selected from LR and 11 features from SVM, and all the unique features from the two runs were used to build the models. With an accuracy of 87.2%, CatBoost achieved the highest score (Table 5) for the selected features. It is worth mentioning that we selected 20 unique variables based on RFE, where most of these variables, were statistically significant (*p*-value $\le$ 0.05).

### Comparison against other methods

Our present study puts forward ML models to differentiate between the diabetic and non-diabetic groups in a cohort from Qatar. Prior research has highlighted the widespread application of ML in healthcare. For instance, in a study of 68,994 individuals with diabetes and healthy individuals from China, the random forest method demonstrated the highest accuracy (ACC = 80.84%) after identifying appropriate features [33]. Another study [34] involving 768 patient records of Pima Indian women with nine attributes showed that SVM and KNN provide the highest degree of accuracy in predicting diabetes. Compared to the other algorithms used in that paper, both algorithms provide 77% accuracy [34]. It is plausible that ML can be used to predict diabetes, but it will require finding appropriate attributes, classifiers, and data mining methods. According to a study [15] conducted in Qatar, retinal images can be used to determine whether a patient has diabetes or not. An accuracy level of over 84% was achieved using a multi-stage convolutional neural network (CNN)-based model DiaNet [15]. There was another study [14] in Qatar which used QBB data to develop machine-learning models to differentiate diabetic patients from non-diabetic participants. Several hundred measurements were analyzed to identify 25 potential risk factors that might help distinguish diabetic patients from non-diabetics. According to the results, HbA1c, Glucose, and LDL-Cholesterol were the most influential risk factors. Classifiers perform nearly the same, with SVM slightly outperforming linear regression (LR) and quadratic discriminant analysis (QDA) at accuracy (0.881) [14]. However, they were able to achieve this accuracy because they include both HbA1c and Glucose measurements as features in ML model, while we did not use these known biomarkers to build ML models since they are already known markers for diabetes and inclusion of those features would improve the prediction accuracy.

It is crucial to highlight that the impact of diabetes on the bone health of patients within the realm of clinical epidemiology remains a subject of debate. While certain studies have shown a potential connection between diabetes and reduced BMD, others have reported BMD levels within the normal range or even increased BMD [31]. In our research, we observed lower BMC and BMD in various anatomical regions among individuals with diabetes when compared to the control group, although these differences did not reach statistical significance. A recent systematic review has also drawn similar conclusions, suggesting a lack of a definitive link between diabetes and the deterioration of bone health [35]. Our study reaffirms these findings, based on the QBB cohort. However, it is imperative to conduct further investigations in clinical settings to delve deeper into the potential connections between diabetes and bone health decline.

### Limitations

This research is limited by the size of the dataset and the number of missing attribute values. Our cohort covered only 500 diabetic patients and 500 control individuals. In addition, we focused exclusively on Qatari nationals, hence the results of this study may not be applicable to other cohorts from different ethnicity without validation. Nevertheless, we expect the results of this study to be applicable to other GCC nations since lifestyle and behavioral characteristics of Qatari nationals are comparable among GCC nationals.

## Conclusion and future works

Diabetes prediction at an early stage is one of the key research areas in healthcare. Clinicians could detect diabetes earlier with the help of a ML-based approach. In this study, ML models were utilized to determine whether an individual will get diabetes at an early stage. ML models predicted more accurate results when combining DXA measurements and clinical data, which indicates the importance of incorporating DXA scan with existing clinical data for the early diabetes detection. Our study highlighted key factors i.e., cholesterol, neutrophil, sodium, chloride, bilirubin, AST, GGT, etc. for the early detection of diabetes . We also showed that the effect of diabetes on bone health over time is not significant. These results showed great promise in detecting prediabetes early on and, as a result, reducing the incidence of diabetes in the region. Our future work will focus on integrating other methods i.e., ensemble-based methods to improve the performance of models for better accuracy. Testing the models on larger datasets may reveal more insights and better prediction accuracy. Considering the clinical significance of HbA1c levels in diabetes management and the heterogeneity within Type 2 diabetes conditions, a regression model predicting HbA1c values could offer a more detailed and clinically relevant outcome which we will focus as part of our near future endeavor.

### Supplementary Information

Supplementary Material 1.

## Data Availability

Data used in this research can be accessed upon the approval from QBB. Please contact takepart@qatarbiobank.org.qa for data access.
